# Surgery versus Active Monitoring in Intermittent Exotropia (SamExo): study protocol for a pilot randomised controlled trial

**DOI:** 10.1186/1745-6215-13-192

**Published:** 2012-10-16

**Authors:** Deborah Buck, Elaine McColl, Christine J Powell, Jing Shen, John Sloper, Nick Steen, Robert Taylor, Peter Tiffin, Luke Vale, Michael P Clarke

**Affiliations:** 1 Institute of Neuroscience, c/o Clinical Trials Unit, Medical School, Newcastle University, Newcastle upon Tyne, UK; 2 Institute of Health & Society, Baddiley-Clark Building, Newcastle University, Newcastle upon Tyne, UK; 3 Royal Victoria Infirmary Eye Department, Newcastle upon Tyne Hospitals NHS Trust, Newcastle upon Tyne, UK; 4 Moorfields Eye Hospital, London, UK; 5 York Hospitals NHS Trust, York, UK; 6 Sunderland Eye Infirmary, Sunderland, UK; 7 Institute of Neuroscience, Medical School, Newcastle University, Newcastle upon Tyne, UK

**Keywords:** Intermittent exotropia, Divergent strabismus, Surgery, Feasibility studies, Pilot study, Randomised controlled trial, Children, Parents, Qualitative research

## Abstract

**Background:**

Childhood intermittent exotropia [X(T)] is a type of strabismus (squint) in which one eye deviates outward at times, usually when the child is tired. It may progress to a permanent squint, loss of stereovision and/or amblyopia (reduced vision). Treatment options for X(T) include eye patches, glasses, surgery and active monitoring. There is no consensus regarding how this condition should be managed, and even when surgery is the preferred option clinicians disagree as to the optimal timing. Reports on the natural history of X(T) are limited, and there is no randomised controlled trial (RCT) evidence on the effectiveness or efficiency of surgery compared with active monitoring. The SamExo (Surgery versus Active Monitoring in Intermittent Exotropia) pilot study has been designed to test the feasibility of such a trial in the UK.

**Methods:**

Design: an external pilot patient randomised controlled trial.

Setting: four UK secondary ophthalmology care facilities at Newcastle NHS Hospitals Foundation Trust, Sunderland Eye Infirmary, Moorfields Eye Hospital and York NHS Trust.

Participants: children aged between 6 months and 16 years referred with suspected and subsequently diagnosed X(T). Recruitment target is a total of 144 children over a 9-month period, with 120 retained by 9-month outcome visit.

Randomisation: permuted blocks stratified by collaborating centre, age and severity of X(T).

Interventions: initial clinical assessment; randomisation (eye muscle surgery or active monitoring); 3-, 6- and 9-month (primary outcome) clinical assessments; participant/proxy completed questionnaire covering time and travel costs, health services use and quality of life (Intermittent Exotropia Questionnaire); qualitative interviews with parents to establish reasons for agreeing or declining participation in the pilot trial.

Outcomes: recruitment and retention rates; nature and extent of participation bias; nature and extent of biases arising from crossover or loss to follow-up; reasons for agreeing/declining participation; variability of cure rates (to inform power calculations for a definitive RCT); completion rates of outcome measures.

**Discussion:**

The SamExo pilot trial will provide important pointers regarding the feasibility of a full RCT of immediate surgery versus deferred surgery/active monitoring. The results of this pilot, including differences in cure rates, will inform the design of a definitive RCT.

**Trial registration:**

ISRCTN44114892

## Background

Strabismus, also known as squint, is an ophthalmic condition in which the eyes are misaligned and therefore look in different directions: that is, one eye looks straight ahead while the other turns either outward (exotropia), inward (esotropia), upward (hypertropia) or downward (hypotropia) [1]. It may be constant, with loss of binocular function, or intermittent, with binocular function when the squint is not present. Squint can occur in children or adults and may have functional, aesthetic and psychosocial consequences [2-16]. For example, teenagers and adults with squint have reported problems with self-esteem, self-image and interpersonal relationships, have met ridicule at school or work, and may attempt to avoid activities that bring attention to their condition or to develop strategies that conceal it [8,10]. Similarly in young children, squint has been linked to lower psychosocial functioning, poorer interpersonal relationships and lower self-esteem [15,16]. It has been shown that children as young as 5 years are significantly more likely to have negative social reactions to peers with strabismus [13,14] and that teachers rate photographs of children with strabismus more negatively than those with straight eyes [15].

Parents may seek treatment for their child’s squint out of concern that abnormal ocular alignment could result in social exclusion and bullying [6]. However, conservative treatment with eye patches [17-19] or glasses may in itself negatively affect the child’s psychosocial well-being and lead to increased bullying and stigmatisation [6,11,20,21]. As Menon et al. [8] report, these problems sometimes remain through to adulthood. Parents of children with squint may themselves experience a negative impact on their own psychological well-being, parental role, family functioning and general functioning [22,23].

Intermittent exotropia [X(T)] is one of the commonest types of childhood strabismus [24,25]. In this condition, one eye intermittently drifts outward. It is possible for X(T) to develop into a constant squint (XT) [24,25] and lead to loss of stereovision and/or the development of amblyopia [18] (reduced acuity in one eye caused by decreased quality visual input during the critical period of development). Typically, X(T) is first spotted in early childhood by parents noticing that their child’s eye is wandering outward as they look at objects in the distance or when they are very tired, inattentive or in bright sunlight.

Conservative treatment options for X(T) include occlusion with eye patches or wearing glasses that stimulate convergence. Eye muscle surgery can also be performed in order to realign the eyes. However, many clinicians and parents opt for an active monitoring approach, that is, they decide to wait and see whether the squint resolves spontaneously or at the very least does not deteriorate. Long-term natural history data are lacking, but there is some indication from observational work that X(T) surgery is more successful than conservative treatment and active monitoring in improving control of the eyes [19]. However, the success of surgery is not guaranteed: it comes with a risk of over-correction whereby the eye alignment converts to a constant convergent deviation (esotropia) with a consequent loss of function in terms of stereovision and the potential to develop amblyopia in young children or diplopia (double vision) in older children. Alternatively, an under-correction may occur with persistence of the X(T). There is also evidence that the effectiveness of surgery declines over time [26]. There is considerable variability in rates of surgery and a lack of consistency in the surgical indications for treatment of X(T) in the UK [18,19]. Here, the majority of children with the condition do not appear to undergo eye muscle surgery within 2 years of presentation and instead are monitored recurrently, with significant loss to clinical follow-up.

Surgical intervention and active monitoring together incur substantial costs to the UK National Health Service (NHS). For example, over 6,000 procedures were performed by the NHS on children under 16 years in 2007/8, of which around 1,000 are estimated to have been performed for X(T). The tariff rate for strabismus surgery is £800, and together with approximately 100,000 clinic visits annually for review of patients with X(T) (at a tariff cost of £120 per new patient and £60 per review: estimated average new:review ratio 1:8), the total cost to the NHS alone is almost £7.5 million annually. With the inclusion of societal and family costs, the management of X(T) is more costly. Our previous cohort study [18,19] has demonstrated not only considerable variability in management between centres, but also a ‘therapeutic inertia’, with many children receiving no active treatment and many eventually defaulting from follow-up, contributing to high DNA (did not attend) rates. Without further research, inefficiencies in the use of NHS and societal resources used to manage X(T) are likely to remain.

The lack of trial-based evidence means that the true effectiveness of treatment in ameliorating or curing the condition is unknown. Moreover, even when surgery is the preferred course of action there is little agreement on whether immediate surgery is more effective than delaying the operation for a specified length of time or until a certain age. A pressing need for carefully planned clinical trials of treatment to improve the evidence base for the management of this condition has previously been identified [27].

The current investigators hope to conduct such a trial, if feasible. Since the recruitment phase in any trial is one of the most challenging [28,29], and given the potential recruitment barriers particularly inherent in paediatric [30-33] or surgical [34] trials, we are first undertaking the SamExo (**S**urgery versus **A**ctive **M**onitoring in Intermittent **Exo**tropia) pilot trial in order to assess feasibility and inform the design and conduct of a full-scale trial ( http://research.ncl.ac.uk/samexo/).

Parents of children with X(T) participating in a previous observational cohort study [The Improving Outcomes in Intermittent Exotropia (IOXT) study] [18,19] were surveyed and their comments taken into account in developing the present study, together with input from the lay group of the Royal College of Ophthalmologists and the patient and public engagement committee of the Newcastle NHS Hospitals Foundation Trust. Comments included the need to carefully explain the potential risks and benefits of a child with X(T) being in the active monitoring arm. Overall, comments were supportive of the need for such a study.

Our ultimate goal is to improve the treatment that children with X(T) receive by evaluating the risks and benefits of surgery in an RCT in which some children, where safe to do so, have surgical treatment deferred. Equally, the trial would enable us to determine whether some children get better spontaneously and to assess the effect of the condition and its treatment on quality of life (QOL). We would also evaluate costs to families and to the NHS and whether surgery represents a good use of the resources available.

The specific objectives of the current SamExo pilot trial are:

1. To determine whether participating centres are likely to recruit a sufficient number of patients to deliver a full trial.

2. To determine whether recruited patients will stay within their allocated groups and complete follow-up in sufficient numbers to deliver the trial.

3. To identify reasons why parents accept or decline participation in the trial.

4. To pilot the procedures involved in the trial including recruitment (giving information and obtaining consent), randomisation, intervention (surgery), masking, outcome measurements, and web-based trial management and data capture systems.

## Methods

### Study design

**Figure 1:**
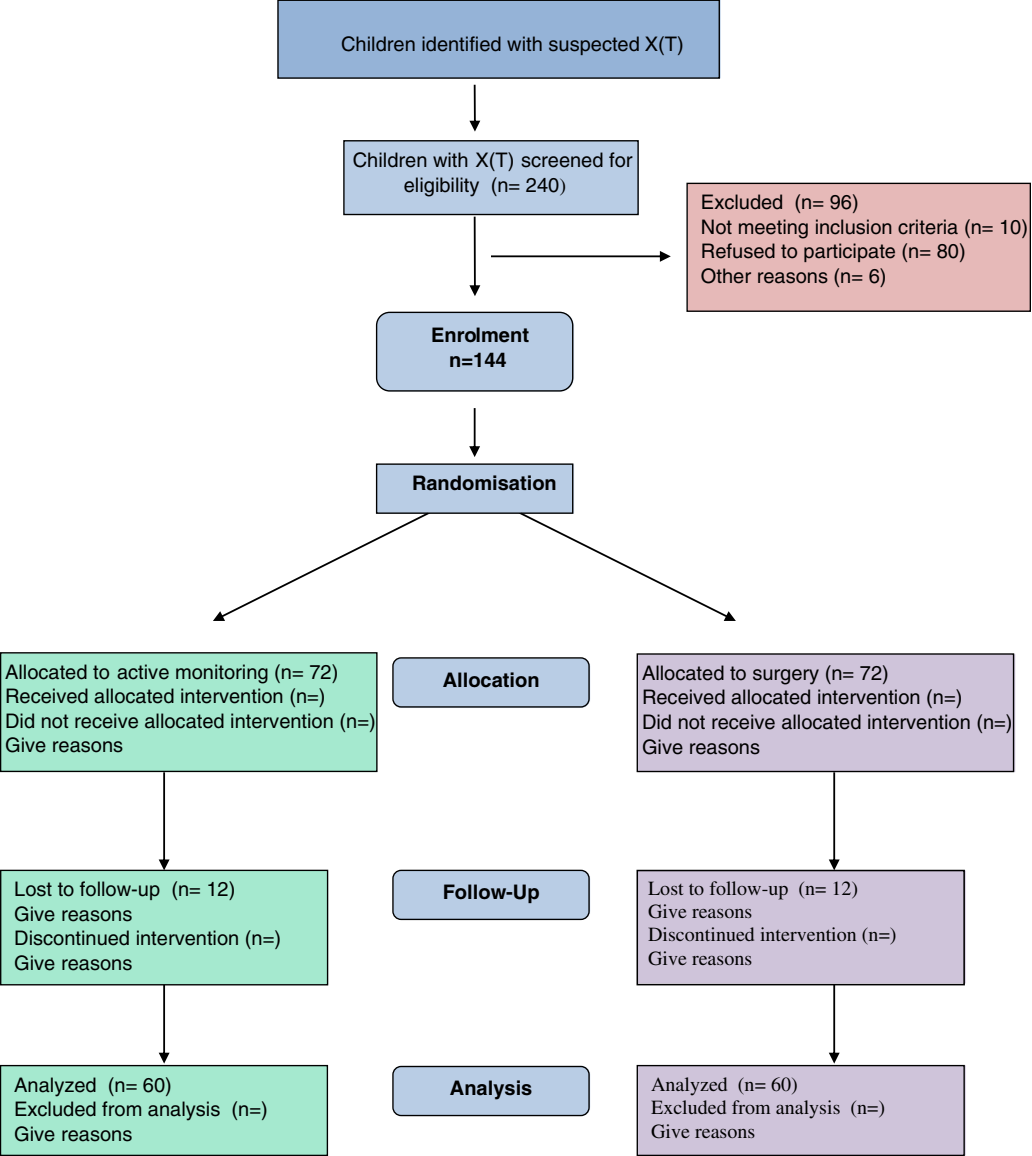
SamExo pilot trial design.

SamExo is a rehearsal pilot randomised controlled trial (RCT) to assess the feasibility of a full RCT of the effectiveness of surgical treatment against active monitoring in X(T) (Figure 1). The trial is being conducted according to recommendations for good practice in pilot studies [35].

### Study setting

Four secondary ophthalmology care facilities at the Newcastle upon Tyne NHS Foundation Trust (coordinating site), Sunderland Eye Infirmary, Moorfields Eye Hospital and York NHS Trust, each of which are large centres with specialist paediatric ophthalmology clinics.

### Participants and sample size

Children aged between 6 months and 16 years referred to the clinics with suspected X(T) from community screening, general practice or other health-care professionals, and subsequently diagnosed with X(T), as well as existing patients fulfilling the eligibility criteria. The parent/guardian provides written informed consent for participation in the study prior to any trial-specific procedures.

A formal power calculation was not performed for this feasibility study. It is not powered to detect a clinically or economically meaningful difference in the primary outcome between the surgical and active monitoring groups. Rather, the aim is to provide robust estimates of the likely rates of recruitment and retention, and to yield estimates of the variability of the primary and secondary outcomes to inform power calculations for a subsequent full-scale RCT. We originally estimated that over a recruitment period of 6 months (subsequently extended to 9 months) across four centres, we would be able to approach 240 patients who met the entry criteria. From their responses we would be able to determine whether the study is acceptable to parents and consequently whether it is possible to recruit patients and follow them up. We would also be able to estimate attrition rates. By approaching 240 children/parents we would be able to estimate the recruitment rate with a standard error no larger than 3.3%. Assuming that half of these children are actually recruited, we will be able to estimate the 6-monthly attrition rate with a standard error no larger than 4.3%.

### Clinical tests

The following routine clinical assessments are undertaken at each visit:

• Binocular single vision (BSV) testing − either stereovision or motor fusion (reflex convergence of the eyes in response to a base out prism);

• Best corrected visual acuity (VA) on an age-appropriate test;

• Measurement of the ocular misalignment using the Alternate Prism Cover Test (APCT);

• Measurement of how well the squint is controlled using the revised Newcastle Control Score (NCS) [36] and the Mayo score [37]. The NCS combines an estimate of observed frequency of the X(T) by parents (home control) with an assessment of the child’s ability to realign the eye following a cover test to induce misalignment (clinic control for distance and near fixation) − possible total NCS scores range from 0–9 (0–3 for home, 0–3 for clinic distance and 0–3 for clinic near control). On the Mayo score, the distance score (0 to 5) is combined with near score (0 to 5) to yield an overall control score ranging from 0 to 10. On both instruments, higher scores are indicative of poorer control.

### Inclusion criteria

• Age between ≥ 6 months and ≤ 16 years

• Evidence of X(T) on the basis of parental history and clinical examination

• No ongoing or planned amblyopia treatment

• VA of 0.500 or better on an age-appropriate logMAR-based test or, where uniocular testing is not possible, central steady maintained fixation when one eye is occluded

• NCS of ≥ 3

• Minimum of 15 dioptres misalignment in the distance

• Presence of near stereopsis documented using the preschool Randot Test if ≥ 3 years of age

• If < 3 years old must be able to overcome a base out prism (10, 15 or 20^)

### Exclusion criteria

• Age over 16 years

• Previous treatment for X(T)

• Constant XT (other than microtropia)

• VA of > 0.500 logMAR in either eye

• X(T) where near misalignment is >10 prism dioptres more than the distance misalignment

• Structural ocular pathology

• Significant neurodevelopmental delay

• Families planning to move out of area

### Recruitment and consent

Children referred from community screening, general practice or other health professionals are clinically assessed in the normal way when they attend their initial outpatient visit. Where appropriate, glasses are prescribed to correct any refractive error. Discussion about the management of the X(T) itself includes providing information about the study and the possibility of taking part. Parents who express an interest are then given written information and subsequently telephoned by a member of the study team to discuss any queries they may have once they have had time to read the documentation and talk things through with their families. All parents are given an appointment on a study recruitment clinic. At the recruitment clinic, eligibility is confirmed by checking the results of the initial routine clinical assessment against the inclusion criteria. Eligible parents are then asked if they would like to enter the study. Informed consent is obtained from those who decide to participate. Participants are also being identified from existing lists of patients who are already under follow-up and fulfil the eligibility criteria; we are adhering to the same information-giving and consent procedures as for new referrals.

The following routine clinical assessments are made in order to confirm the patient’s eligibility for the trial:

• BSV testing;

• Best corrected VA on an age-appropriate test;

• Measurement of the ocular misalignment using the APCT;

• Measurement of how well the squint is controlled using the NCS and Mayo scores.

Eligible families who do not wish to take part in the trial are asked whether they would consent to come back for a follow-up appointment after 9 months and allow us to use routine clinical data from that visit and their initial visit. Data from those who agree to this will be used to verify whether the recruited group is representative and to determine how many of those eligible but not recruited went on to have surgery during that 9-month period.

### Interventions

#### Clinic appointments

**Figure 2:**
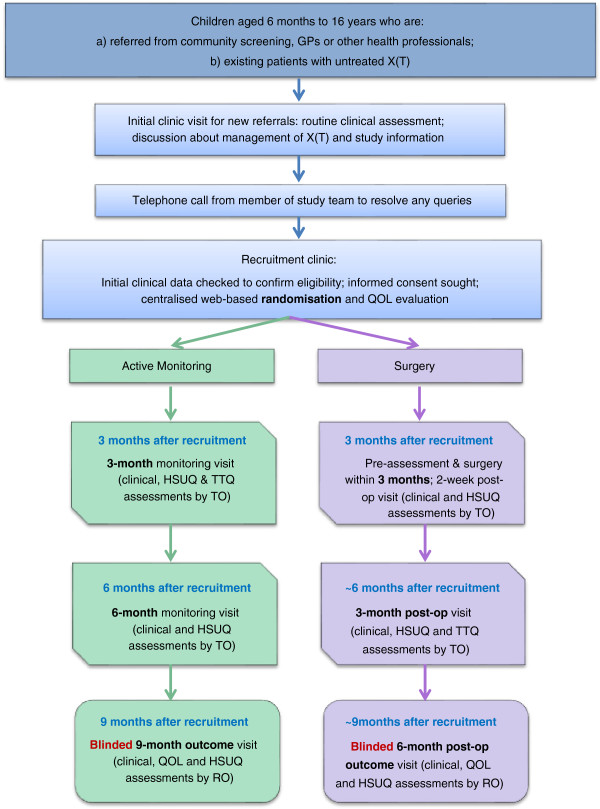
**Summary of study visits, assessments and interventions.** Abbreviations: HSUQ = health services use questionnaire; QOL = quality of life; RO = Research orthoptist; TO = treatment orthoptist; TTQ = time and travel questionnaire.

Figure 2 illustrates the schedule of study visits and corresponding assessments for the active monitoring and surgery arms. The assessments involve routine clinical measurements together with the evaluation of QOL using the Intermittent Exotropia Questionnaire (IXTQ) [38], a health services use questionnaire (HSUQ) and a time and travel questionnaire (TTQ). Children in the active monitoring group will be offered surgery if a constant strabismus appears to be developing or parents request surgery and the responsible clinical team agrees that this is appropriate.

#### Eye muscle surgery

Surgery is performed by the local principal investigator (PI), or delegated deputy, according to agreed surgical formulae tailored to the clinical characteristics of the strabismus and the usual practice of the surgeon. Principles involved in the surgical treatment of children in the study have been agreed as follows:

• General anaesthesia

• Bilateral lateral rectus recession surgery to be performed for true distance exotropia

• Unilateral recess/resect surgery to be performed for other types of exotropia

• Standard sterile preparation of the operative site

• Conjunctival incisions

• Standard isolation and cleaning of muscle to be operated

• Muscle secured with 6/0 vicryl suture

• Amount of recession/resection assessed on the basis of the maximum distance angle according to table, modified according to standard practice of surgeon

• Measurement of amount of muscle adjustment to be checked post placement of scleral sutures

• Conjunctival incisions closed with vicryl sutures

• Topical anaesthetic and antibiotic drops given at the end of procedure

A surgical table (Table 1) is being used with modification as appropriate to determine the amount of eye muscle movement to be performed depending upon the size of the angle of exotropia. Surgical technique is carefully recorded and monitored during the pilot with a view to standardising surgical technique, as far as it is possible to do so, in a full trial and provide a clear description of the intervention in subsequent reports.

**Table 1:** Surgical table for use in the SamExo pilot trial

**Angle of deviation (dioptres)**	**Lateral rectus recession (mm)**	**Medial rectus resection (mm)**	**Bilateral lateral rectus recession (mm)**
20	4	3	4.5
25	5	4	5
30	5.5	4	6
35	6.5	4.5	6.5
40	7	4.5	7
50	8	4.5	8

#### Primary outcome visit

The final assessment (9-month outcome) will be conducted by a research orthoptist (RO) who is masked to the allocation of the child and has not otherwise been in contact with children enrolled in the study. The parent and child will be requested not to reveal the group allocation of the child to the RO prior to the assessment. While children have noticeably red eyes immediately following eye muscle surgery, it is recognised that this redness resolves within 6 weeks when this is a first procedure. Residual scarring of the conjunctiva following eye muscle surgery will be inconspicuous by 6 months.

#### Parent interviews

We are gathering qualitative data (primarily through telephone interviews) from parents to explore their reasons for either accepting or declining participation in the pilot study, their thoughts on randomisation and the study information received, and any ideas they may have for improving the trial. These data will inform the design of a full RCT. Whilst the principles of informed consent mean that individuals are not obliged to give a reason for their decision if they do not want to, we are inviting parents to take part in a telephone interview if they agree to this level of involvement. Without this information, it is difficult to see how the research design can be improved to make it more acceptable. These interviews are being conducted by a university researcher who is entirely separate from the clinical team, emphasising that parents’ decisions not to participate have been respected and reassuring that no attempt is being made to change their minds.

### Randomisation

Randomisation is in permuted blocks stratified by collaborating centre, age and severity of X(T) as measured by the NCS. A blocked allocation (permuted random blocks of variable length) system is being used to allocate patients to the two groups in a 1:1 ratio to intervention (surgery) and control (active monitoring) groups. Randomisation is administered using a centralised, password-protected web-based system, which is managed by the Newcastle Clinical Trials Unit (NCTU). The PI at site, or individual with delegated authority, enters the patient ID, initials and the stratifying variables, which then return the allocation status. Participants are informed of their group allocation and given the appropriate Group Allocation Information sheet.

### Allocation concealment

Since surgery is the intervention of interest it is not possible to mask participants or parents to their group allocation. Masking of investigators is being achieved by the designation of a treatment orthoptist (TO) and a RO at each site. TOs cannot be masked to the group allocation of participants since they will conduct all assessments other than the outcome assessment and deal with queries from parents/children during the course of the trial. Clinical examination at the primary outcome visit will be carried out by the ROs who will be unaware of treatment group allocation. At this final study visit, the success of the masking will be assessed by asking the outcome assessor: “Do you think the patient has had surgery or not? Why do you think this?” Their responses will be recorded on a separate form.

### Proposed outcome measures

For children with X(T), and their parents, the most relevant outcome from intervention is the restoration of normal eye alignment, with associated cosmetic and functional benefits. The primary outcome in a full-scale trial will therefore be the difference in the cure rate of X(T) between the surgical and actively monitored group; this is also the primary outcome for which data will be collected in the current pilot in order to inform sample size calculations for a definitive RCT. Cure will be defined as:

• a control score (NCS) of 0 (misalignment never noticed by parents, no observable deviation on cover test)

• demonstrable near stereoacuity in children over 3 years of age

Secondary outcomes include age-specific QOL assessments, median scores of control of exotropia assessed by parental report and clinical components of the NCS and the Mayo Score, rates of amblyopia, use of health-care resources, NHS costs, costs to families accessing the treatments being evaluated and incremental cost per cured patient (with cure as defined by the primary outcome), and a cost-consequences analysis based on the incremental cost with respect to changes in all relevant outcomes where possible.

The key outcomes of this pilot study are:

• data on the variability of the primary and secondary outcome measures;

• rates of participant recruitment and randomisation;

• nature and extent of participation bias;

• rates of cross-over and retention of recruited participants;

• nature and extent of biases arising from cross-over or loss to follow-up.

An initial recruitment rate of >60% and a retention rate of >70% will be considered necessary to indicate feasibility of a full-scale RCT.

Our intention in the full trial would be to conduct a cost-effectiveness analysis based on incremental cost per cured patient (as defined by the primary outcome) and a cost-consequences analysis based on incremental cost with respect to changes in all relevant outcomes where possible, including the QOL measure and different clinical measures. In the pilot study, we will rehearse this cost-effectiveness analysis to inform the study hypothesis and the analysis plan for the definitive trial. We will also assess the ease of collecting information on outcome and costs needed for the health economics analysis.

With respect to collecting costs data, we will pilot the TTQ and HSUQ that capture patient costs and the NHS costs. We will assess the response and completion rates of these instruments. Patient costs will include travel costs for accessing NHS primary and secondary care; time costs of travelling and attending NHS primary and secondary care; and self-purchased health-care and related management costs. NHS costs will comprise use of health-care resources in both primary and secondary care. Total costs consisting of patient costs and the NHS costs will be compared between the randomised interventions.

### Statistical methods

For primary outcomes, t-tests and Wilcoxon tests will be used to compare the age and severity of X(T) of those participants retained with any withdrawing from the trial and to compare those who consent with those eligible but refusing to participate. A chi-square test will be carried out to assess the variability of the primary outcome (difference in cure rate at the final assessment between the surgery and active monitoring arm).

For secondary outcomes, non-parametric testing (Wilcoxon) will be used to compare change over time in median control scores within the two groups between baseline and 9-month follow-up (Mann-Whitney tests will examine any differences between the groups at baseline and 9 months); rates of development of amblyopia in the two groups will be determined by monitoring visual acuity at the 3-, 6- and 9-month assessments. For the QOL measurements, we are primarily concerned with response and completion rates for these instruments in both groups; in addition, their validity will be assessed by matching individual pre- and post-treatment scores to the post-treatment primary outcome using t-tests.

Statistical significance will be set at *p* < 0.05. Data will be analysed with the SPSS statistical package.

### Trial governance

Sunderland Research Ethics Committee (REC) has given the SamExo pilot trial a favourable ethical opinion (reference 10/H0904/57). The Newcastle upon Tyne NHS Hospitals Foundation Trust is acting as the trial sponsor. A Trial Management Group is overseeing the overall conduct and progress of the study and provides day-to-day support to collaborating sites. Training has been given to collaborators through investigator meetings, site initiation visits and routine monitoring visits. Quality control is maintained through adherence to the study protocol, Standard Operating Procedures (SOPs), principles of good clinical practice, research governance and clinical trial regulations. As agreed by the sponsor a Trial Steering Committee (TSC), which includes a parent representative, is adopting the joint roles of TSC and Data Monitoring & Ethics Committee (DMEC), with independent members meeting in closed session to fulfil the DMEC role.

### Potential risks and adverse events

This is a low-risk trial and major safety data are not anticipated. The major identified risk for participants is that those in the active monitoring arm may experience a natural deterioration in their clinical condition. This risk will be managed by 3-monthly assessments of the child for the duration of the study. Children will be offered treatment if there is a significant deterioration, or if the parent wishes to discontinue monitoring in favour of eye muscle surgery and the clinical team agrees that surgery is appropriate following a discussion of the risks and benefits, based on currently available information. Participation may generate anxiety for some parents and children [39]: this risk will be managed by providing ready access to sources of advice and support (for example the TO). Study information provided to parents and participants includes an account of existing knowledge of the natural history and current management of X(T). Parents are informed that their children will be regularly assessed and encouraged to report concerns about their child’s X(T) to the TO who will take these to the local PI. The latter will arrange for eye muscle surgery if the child meets the usual criteria for surgery. Parents will be encouraged to report concerns about the conduct of the study to the Trial Steering Committee, Sponsor and Funder.

Adverse event reporting following surgery will be undertaken in accordance with the National Research Ethics Service (NRES) guidelines for adverse event reporting in non-CTIMP trials. A serious adverse event (SAE) in this pilot trial is defined as an untoward occurrence that:

• results in death

• is life-threatening

• requires hospitalisation or prolongation of existing hospitalisation

• results in persistent or significant disability or incapacity

• is otherwise considered medically significant by the investigator

Expected adverse events, both common and rare after X(T) surgery, are listed in Table 2. If a patient experiences an SAE it will be reported to Sunderland REC when, in the opinion of the chief investigator, the event was ‘related’: that is, resulted from administration of any of the research procedures; and ‘unexpected’: that is, the type of event is not listed in the protocol as an expected occurrence. Any SAEs will be reported to the NCTU within 24 h of the PI learning of its occurrence. Reports of related and unexpected SAEs will be submitted to the REC within 15 days of the chief investigator becoming aware of the event, using an NRES ‘report of serious adverse event’ form.

**Table 2:** Expected adverse events during the SamExo pilot trial

**Procedure**	**Adverse event:**	
	**Common & well understood consequences of treatment**	**Rare events**
Perforation of the globe	Occurring within 24 h	Occurring after 24 h
Intraocular infection	Occurring within 2 weeks	Occurring after 2 weeks
Lost or slipped muscle	Occurring within 1 month	Occurring after 1 month
Scleritis	Occurring within 1 month	Occurring after 1 month
Becoming constant XT	Occurring within 9 months	Occurring after 9 months
Persistent over-correction	Occurring within 9 months	Occurring after 9 months
Re-operation for under- or over-correction	Occurring within 9 months	Occurring after 9 months

## Discussion

The optimum management of X(T) in children is contentious, with some clinicians recommending early surgery, some preferring to delay and others choosing to actively monitor. The lack of robust evidence to guide management and the poorly understood natural history of this condition renders it difficult for clinicians to offer clear advice to parents. Consequently, many children are observed without any treatment for extended periods because of the uncertainty surrounding the best course of action. The need for evidence from randomised studies has been acknowledged for some time now [27], and this is the first attempt, as far as we are aware, to explore the potential for a RCT of surgery versus active monitoring in childhood X(T). We felt it would be inappropriate to undertake a full-scale study involving large numbers of participants and centres before first conducting a feasibility study. The SamExo pilot study will provide important pointers regarding how acceptable a RCT of immediate surgery versus deferred surgery/active monitoring will be to participants and clinicians.

The recruitment phase will give us a good indication of the proportion of eligible children/parents who would sign up to a full trial, the likelihood of them withdrawing from it and also an idea of how many we would need to approach over a given duration to achieve a specified sample size.

Equally pivotal will be the reasons why parents agree or decline the pilot trial, their perceptions of the need for and purpose of randomisation, their views on the nature of the information they received, and suggestions for improving future trials and refining the research question. Findings from these qualitative interviews will not only inform the design of a possible full trial but may also help investigators planning studies of surgical interventions, trials of other children’s eye conditions or paediatric trials in general.

Potential ethical issues include the fact that some of the children in the SamExo pilot study will be asked to wait up to 9 months before being offered surgery. We feel that this can be justified as our previous work has shown that less than 8% of children with X(T) had surgery within the first year following initial assessment when treated in a routine setting [18]; in some centres, no children had surgery within 2 years of diagnosis [19]. Children who have very mild X(T) are not being entered into the trial, even though our earlier research found that some of these children did have surgery. Similarly, we are not recruiting children who appear to be at imminent risk of developing a constant squint. Children who are in the active monitoring arm are being regularly assessed and will be offered treatment if they develop, or seem to be at risk of developing, a constant squint. With increased rates of surgery, we may see increased rates of re-operations, persistent over-correction and other surgical complications.

## Trial status

Recruitment to the SamExo pilot trial commenced in September 2011 and is ongoing at the time of manuscript submission. Recruitment was originally scheduled to end in February 2012 but we have extended this phase up to 31 May 2012. (The total duration of the trial is unchanged.) This extension to recruitment was recommended by the TSC in January 2012 and agreed upon by the funders in light of early indications that fewer screened patients were actually eligible for the study and fewer eligible patients had thus far agreed to participate than was predicted.

## Abbreviations

BSV: Binocular stereovision; DMEC: Data Monitoring & Ethics Committee; HSUQ: health services use questionnaire; IOXT: The Improving Outcomes in Intermittent Exotropia study; IXTQ: Intermittent Exotropia Questionnaire; NCS: Newcastle Control Score; NCTU: Newcastle Clinical Trials Unit; NRES: National Research Ethics Service; PI: principal investigator; QOL: quality of life; REC: research ethics committee; RO: research orthoptist; SAE: serious adverse event; SamExo: The Surgery against Active Monitoring in Intermittent Exotropia pilot trial; TO: treatment orthoptist; TSC: trial steering committee; TTQ: time and travel questionnaire; VA: visual acuity; X(T): intermittent exotropia.

## Competing interests

The authors declare that they have no competing interests.

## Authors’ contributions

DB drafted the manuscript and contributed to the study design and funding acquisition, and will undertake data monitoring, qualitative work and data analysis. MPC, JS (Sloper), RT and PT conceived the study and contributed to the study design and funding acquisition, and will oversee delivery of the intervention. MPC is the chief investigator. EM contributed to the study design and funding acquisition, and supervised the involvement of the Newcastle Clinical Trials Unit. CJP contributed to the study design, ethics committee and R&D approvals, UKCRN registration, protocol development and creation of case report forms. JS (Shen) and LV contributed to the study design (health economics aspects) and will undertake health economics analysis. NS contributed to the study design (statistical aspects) and will undertake statistical analysis. All authors have commented upon drafts of the paper and have given final approval to this version.

## Authors’ information

Submitted on behalf of the SamExo Study Group.
